# Transient perivascular inflammation of the carotid artery; a rare cause of intense neck pain

**DOI:** 10.1259/bjrcr.20190014

**Published:** 2019-11-15

**Authors:** Yasith Mathangasinghe, Radika Udayangani Karunarathne, Udari Apsara Liyanage

**Affiliations:** 1 Department of Anatomy, Faculty of Medicine, University of Colombo, Sri Lanka; 2 Department of Radiology, Nawaloka Hospital Colombo, Sri Lanka

## Abstract

Carotidynia or Transient Perivascular Inflammation of the Carotid Artery (TIPIC) syndrome is a rare cause of atypical neck pain. Exact aetiopathogenesis of this clinical entity is poorly understood. A 43-year-old female presented with progressively increasing right side neck pain of 3 days duration associated with focal tenderness over the right carotid pulse corresponding to the level of upper border of thyroid cartilage. Her inflammatory markers were not elevated. An ultrasound scan revealed increased echogenicity surrounding the distal common carotid artery, obliteration of the perivascular tissue planes with preserved doppler flow pattern. MRI showed soft tissue thickening around the distal common carotid artery, carotid bulb and proximal external carotid artery on right side of the neck corresponding to sonographic findings with gadolinium enhancement. A diagnosis of TIPIC syndrome was made and she was started on celecoxib. Pain completely subsided within 2 weeks. In conclusion, TIPIC syndrome is a rare differential diagnosis of neck pain. It is caused by a transient perivascular inflammation of the carotid artery. A high degree of suspicion is necessary for the diagnosis. Imaging is the gold standard investigation for the diagnosis of TIPIC syndrome. It is a self-limiting pathology and often responds rapidly to nonsteroidal anti-inflammatory drugs.

## Introduction

In 1927, Fay described a clinical entity characterized by atypical neck pain radiating to the head associated with focal tenderness over the carotid artery, named carotidynia.^1^ In 1988, carotidynia was first considered as a distinct clinical entity from idiopathic neck pain, and was introduced in the International Classification of Headache Disorders as an atypical headache syndrome.^2^ According to this classification, the following four criteria needed to be met to confirm the diagnosis of carotidynia: (a) unilateral neck pain that may radiate to neck; (b) presence of focal tenderness over the carotid artery, oedema or increased pulsation; (c) absence of a structural lesion; and (d) spontaneous recovery within 14 days of the onset of symptoms. Nonetheless, carotidynia was subsequently excluded from this classification in 2004 due to controversial evidence regarding the diagnostic criteria.^3–5^ As opposed to a discrete diagnosis, carotidynia was considered as a non-specific symptom of diseases such as vasculitis, carotid dissection, sialadenitis, trigeminal neuralgia and oropharyngeal infections.^6–9^ However, recent evidence of characteristic radiological findings associated with carotidynia suggest it an isolated diagnosis.^10^ Currently, this clinical pain syndrome is termed Transient Perivascular Inflammation of the Carotid Artery or TIPIC syndrome.^11,12^ There are no published data on the prevalence of this rare and underdiagnosed disorder. In the reported cases, it has a slight female preponderance^11,12^ with the highest incidence in fifth and sixth decades of life.^11,12^ This isolated pathology is believed to be caused by a transient inflammatory process of the vessel wall,^13^ particularly in the adventitia, and the pericarotid tissues.^14^ However, the exact aetiopathogenesis is not well understood and up to date and there is a continuous debate if the two entities, carotodynia and TIPIC syndrome are the same.

## Case report

**Figure 1.**
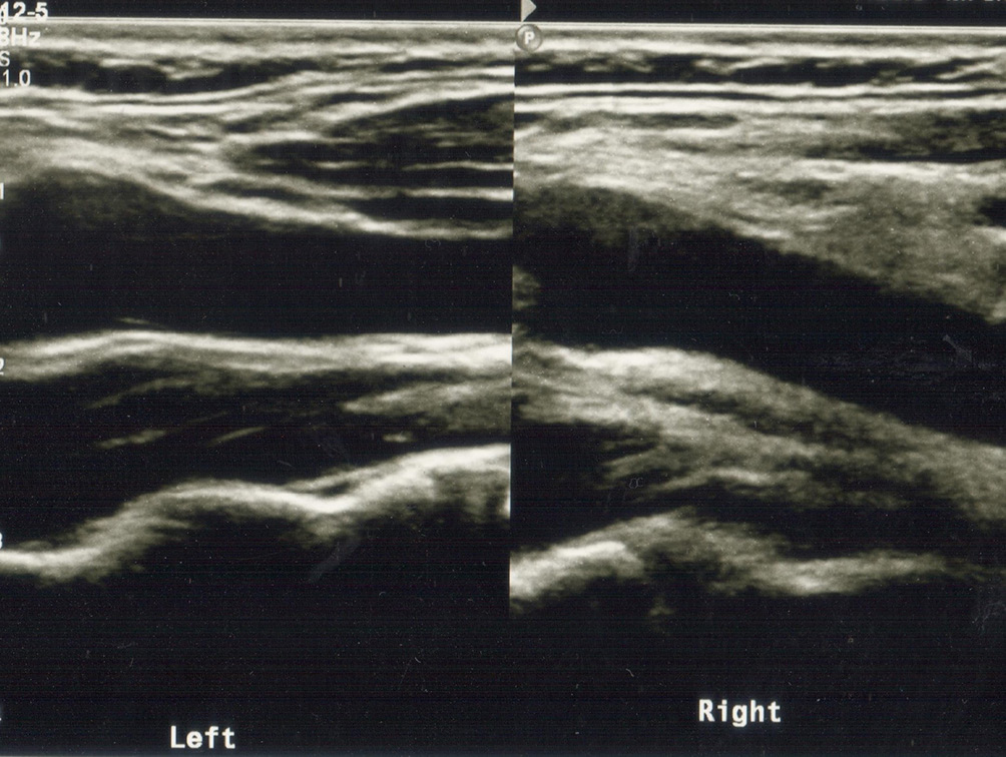
Ultrasonogram of the right side of the neck showing increased echogenicity in the perivascular tissue of the distal common carotid artery and the carotid bulb region with obliteration of normal tissue planes. Normal sonographic appearances of the left carotid artery (left side of the image).

A 43-year-old female presented with progressively increasing right side-neck pain of 3 days duration which was not responding to paracetamol. There was no preceding upper respiratory tract infection or a history of trauma. Examination revealed tenderness on right-side of the neck with mild right-side cervical lymphadenopathy. The complete blood count showed a mild thrombocytopenia and eosinophilia (white cell count - 6.35 × 10^9^ L^−1^, neutrophils - 57.1%, lymphocytes - 29.6%, eosinophil - 4.4%, platelets - 134 × 10^9^ L^−1^). Her C-Reactive Protein level was 3.6 mg L^−1^. Erythrocyte sedimentation rate was 20 mm in the first hour. Due to the intensity of pain, an ultrasound scan of the neck (USG) was performed to look for any suppurative lymphadenopathy. USG reported only a few prominent lymph nodes with otherwise normal morphology at Level II of the neck suggestive of reactive lymphadenopathy. The patient was started on oral Co-amoxiclav and Metronidazol suspecting a dental infection as her last molar tooth was unerupted and a dental referral was planned. Celecoxib was prescribed as the pain was not responding to paracetamol. Her neck pain responded to medication, but disabling intense throbbing pain recurred in-between administration of celecoxib causing patient anxiety. After 4 days of antibiotics, as the pain did not resolve, the patient was clinically reassessed to exclude an alternative pathology. A focal tender point was identified over the right carotid pulse corresponding to the level of upper border of thyroid cartilage querying the possibility of a rare TIPIC syndrome. There were no masses on palpation or bruits on auscultation. A focused second-look ultrasound scan of the neck using 7.5 MHz linear array transducer revealed increased echogenicity mostly of the anterior and lateral aspects of distal common carotid artery, carotid bulb and proximal external carotid artery on right side of the neck. Perivascular tissue planes were obliterated when compared to the normal left side (Figure 1). A focal or diffuse carotid wall thickening was not seen. There were no plaques or luminal stenosis and normal arterial Doppler flow pattern was preserved in carotid arteries bilaterally. The abnormal sonographic findings corresponded to the maximum point of clinical tenderness in the neck. The left side of the neck was normal.

**Figure 2.**
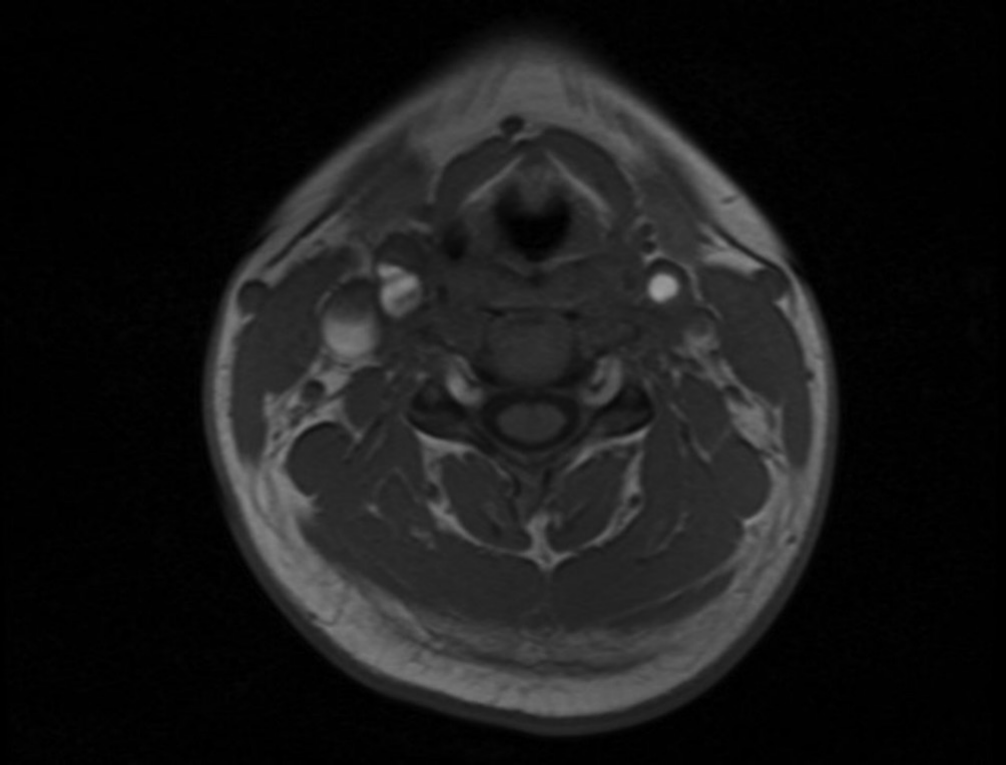
*T*_1_ weighed MR image of the neck showing hypointense perivascular thickening around right carotid bulb.

**Figure 3.**
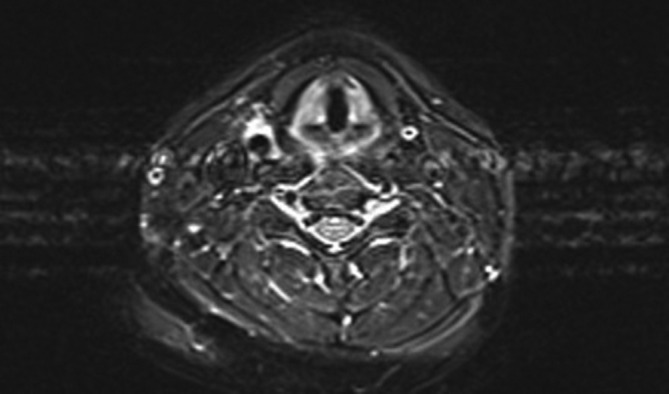
*T*_2_ weighed MR image of the neck showing hyperintense perivascular thickening around distal right common carotid artery.

Diagnosis of TIPIC syndrome was suggested and the patient underwent MRI of the neck. MRI showed soft tissue thickening around the distal common carotid artery, carotid bulb and proximal external carotid artery on right side of the neck corresponding to sonographic findings. Perivascular tissue posterior to the carotid artery were relatively spared. The involved soft tissue appeared hypointense in *T*_1_ weighted sequence (Figure 2) and hyperintense in *T*_2_ weighted sequences (Figure 3) with avid enhancement after intravenous gadolinium administration ). There was no evidence of carotid artery aneurysm or dissection. Immunological screening to look for any connective tissue disease showed normal levels of Anti Nuclear Cytoplasmic (cANCA and pANCA) and Anti Nuclear Antibody levels, and a confident diagnosis of TIPIC syndrome was made. Nonsteroidal anti-inflammatory drugs (NSAIDs) were continued but with poor compliance due to gastric irritation, and the neck pain completely resolved gradually about 2 weeks from the onset of neck pain. Follow-up ultrasound scan of the neck performed 6 weeks after onset of symptoms showed normal echogenicity of previously abnormal perivascular tissues indicating resolution of inflammation. Perivascular tissue planes were normal and similar to the left side in the follow-up scan. Patient did not relapse on clinical follow-up up to 3 months.

**Figure 4.**
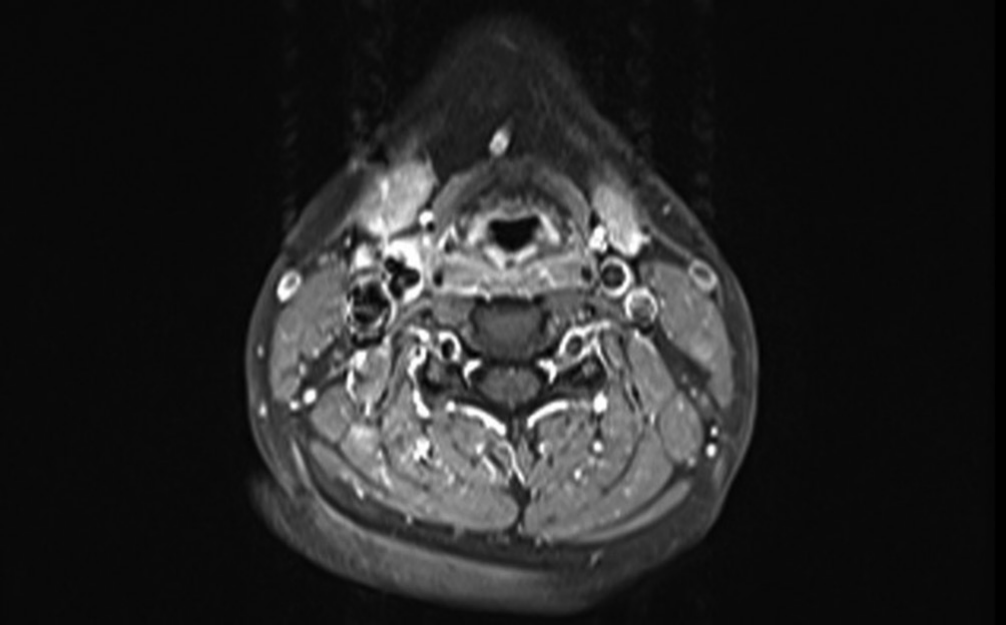
Post contrast *T*_1_ weighted MR image of the neck showing pericarotid enhancement at the level of bifurcation of carotid artery.

## Discussion

TIPIC syndrome typically presents as intense unilateral neck pain with focal tenderness over the carotid pulse at the level of the bifurcation of the common carotid artery.^15,16^ Referred pain in the head and neck region can occur as a result of stimulation of pericarotid sympathetic plexus by the perivascular inflammatory process.^17^ Transient neurological symptoms such as vertigo and diplopia are also reported to be associated with TIPIC syndrome.^12^

Pathogenesis of carotidynia is poorly understood since this entity is essentially a clinical and a radiological diagnosis without the necessity of obtaining biopsies.^14,18^ Upton and colleagues described histological changes of carotidynia for the first time, on a patient undergoing elective carotid endarterectomy for carotid atherosclerosis and who developed carotidynia several days before the elective admission.^13^ There was a predominantly lymphocytic proliferation with scattered neutrophils in the vessel wall. Early fibrosis and vascular proliferation were also evident suggesting that carotidynia is largely a chronic inflammatory process.

As with our case, inflammatory markers are usually not elevated^16,19^ although some authors have reported mild increase in ESR.^13,20^ Imaging is by far the best positive diagnostic tool^12^ as the perivascular inflammatory changes and vessel wall changes are depicted on cross-sectional imaging. Wall change in the carotid artery is shown by ultrasonography as hypoechoic focal mural thickening at the level of carotid artery bifurcation corresponding to the point of maximum tenderness.^15,21^ Generally, this wall thickening is not accompanied by luminal narrowing.^21^ Nevertheless, Abrahamy et al reported carotid arterial luminal narrowing in patients with carotidynia.^15^ However, irrespective of the luminal narrowing, the blood flow within the carotid arteries is not affected.^15,21^ In our case, USG did not reveal any focal mural thickening of the carotid artery as opposed to many case reports,^15,21^ but there was evidence of inflammatory change in the perivascular space corresponding to the maximum point of clinical tenderness over the carotid pulse. The present case shows that although sonographic changes may be obvious to the radiologist in the presence of clinical suspicion the diagnosis can be missed on initial sonographic examination for nonspecific neck pain in the absence of clinical suspicion of TIPIC syndrome which can lead to delayed diagnosis and high level of patient anxiety. Although USG is adequate to establish the diagnosis in the majority, high resolution MRI plays a pivotal role in doubtful cases.^22^ MRI typically shows thickened vessel wall with increased contrast uptake^21^ and pericarotid infiltration.^12^ Contrast-enhanced CT (CECT) may show similar features.^23,24^ Although imaging points to perivascular inflammatory change, TIPIC syndrome is considered largely a diagnosis of exclusion: sinister pathologies related to carotid arteries such as arterial dissection,^15^ aneurysm,^16^ large vessel vasculitis^11^ and autoimmune diseases^12^ need to be ruled out before a confident diagnosis of a transient pericarotid inflammation is made. Such alternative conditions were ruled out in our patient confirming the clinically and radiologically suggested rare diagnosis of TIPIC syndrome.

To date, there are no guidelines as per the treatment of TIPIC syndrome, but NSAIDS are commonly used to accelerate recovery.^11,15,16,21^ Less frequently a short course of steroids may be administered, especially in NSAID non-responding cases.^15,16,21,25^ Very rarely antibiotics were prescribed to treat patients with carotidynia.^15^ Heat therapy may help in alleviating pain in most of the cases.^16^ This clinical entity is essentially described as a transient self-limiting pathology lasting several weeks to a month in the majority.^12,16^ In a recent case report, the follow up positron emission tomography (PET-CT) performed on day 21 of illness demonstrated no residual inflammation.^21^ Nevertheless, this disease is reported to have a recurring sequalae in a minority of patients.^26,27^ Interestingly, it is not clear which speciality should take over the management of these patients. The indexed case was discussed with rheumatologist, neurologist and vascular surgeon creating a dilemma for the general practitioner on management after diagnosis. This further emphasizes the necessity of medical practitioners to be aware of this condition and to have a high degree of suspicion when treating patients with atypical unilateral neck pain.

## Conclusions

Carotidynia or TIPIC syndrome is a rare differential diagnosis of neck pain. It is caused by a transient perivascular inflammation of the carotid artery. A high degree of suspicion is necessary for the diagnosis or diagnosis can be delayed leading to patient anxiety. Imaging is the gold-standard investigation for the diagnosis of TIPIC syndrome. It is a self-limiting pathology and often responds rapidly to NSAIDs. However, TIPIC syndrome is known to recur in some patients.

## Learning points

- Carotidynia or TIPIC syndrome is a rare differential diagnosis of neck pain.
- This is believed to be caused by a transient inflammatory process of the vessel wall and pericarotid tissue.
- Increased echogenicity and soft tissue thickening surrounding the carotid artery at the point of maximum tenderness are classical findings in ultrasound and MRI respectively.
- It is a self-limiting pathology and often responds rapidly to NSAIDs.
